# The current global situation for tuberculous meningitis: epidemiology, diagnostics, treatment and outcomes

**DOI:** 10.12688/wellcomeopenres.15535.1

**Published:** 2019-11-05

**Authors:** James A Seddon, Lillian Tugume, Regan Solomons, Kameshwar Prasad, Nathan C Bahr, Tuberculous Meningitis International Research Consortium, Rob E. Aarnoutse, Rob E. Aarnoutse, Suzanne T. B. Anderson, Suzanne T. B. Anderson, Nathan C. Bahr, Nguyen D. Bang, David R. Boulware, Tom Boyles, Lindsey H. M. te Brake, Satish Chandra, Felicia C. Chow, Fiona V. Cresswell, Reinout van Crevel, Angharad G. Davis, Sofiati Dian, Joseph Donovan, Kelly E. Dooley, Anthony Figaji, A. Rizal Ganiem, Ravindra Kumar Garg, Diana M. Gibb, Raph L. Hamers, Nguyen T. T. Hiep, Darma Imran, Akhmad Imron, Sanjay K. Jain, Sunil K. Jain, Byramee Jeejeebhoy, Jayantee Kalita, Rashmi Kumar, Vinod Kumar, Arjan van Laarhoven, Rachel P-J. Lai, Abi Manesh, Suzaan Marais, Vidya Mave, Graeme Meintjes, David B. Meya, Usha K. Misra, Manish Modi, Alvaro A. Ordonez, Nguyen H. Phu, Sunil Pradhan, Kameshwar Prasad, Alize M. Proust, Lalita Ramakrishnan, Ursula Rohlwink, Rovina Ruslami, Johannes F. Schoeman, James A. Seddon, Kusum Sharma, Omar Siddiqi, Regan S. Solomons, Nguyen T. T. Thuong, Guy E. Thwaites, Ronald van Toorn, Elizabeth W. Tucker, Sean A. Wasserman, Robert J. Wilkinson

**Affiliations:** 1 Department of Infectious Diseases, Imperial College London, London, W2 1PG, UK; 2 Desmond Tutu TB Centre, Department of Paediatrics and Child Health, Stellenbosch University, Cape Town, South Africa; 3 Infectious Diseases Institute, Makerere University, Kampala, Uganda; 4 Department of Paediatrics and Child Health, Stellenbosch University, Cape Town, South Africa; 5 Department of Neurology, All India Institute of Medical Sciences, New Delhi, India; 6 Department of Infectious Diseases, University of Kansas, Kansas City, KS, USA

**Keywords:** Tuberculosis, Meningitis, Tuberculous meningitis, diagnosis, TBM

## Abstract

Tuberculous meningitis (TBM) results from dissemination of *M. tuberculosis* to the cerebrospinal fluid (CSF) and meninges. Ischaemia, hydrocephalus and raised intracranial pressure frequently result, leading to extensive brain injury and neurodisability. The global burden of TBM is unclear and it is likely that many cases are undiagnosed, with many treated cases unreported. Untreated, TBM is uniformly fatal, and even if treated, mortality and morbidity are high. Young age and human immunodeficiency virus (HIV) infection are potent risk factors for TBM, while Bacillus Calmette–Guérin (BCG) vaccination is protective, particularly in young children. Diagnosis of TBM usually relies on characteristic clinical symptoms and signs, together with consistent neuroimaging and CSF parameters. The ability to confirm the TBM diagnosis via CSF isolation of *M. tuberculosis *depends on the type of diagnostic tests available. In most cases, the diagnosis remains unconfirmed. GeneXpert MTB/RIF and the next generation Xpert Ultra offer improved sensitivity and rapid turnaround times, and while roll-out has scaled up, availability remains limited. Many locations rely only on acid fast bacilli smear, which is insensitive. Treatment regimens for TBM are based on evidence for pulmonary tuberculosis treatment, with little consideration to CSF penetration or mode of drug action required. The World Health Organization recommends a 12-month treatment course, although data on which to base this duration is lacking. New treatment regimens and drug dosages are under evaluation, with much higher dosages of rifampicin and the inclusion of fluoroquinolones and linezolid identified as promising innovations. The inclusion of corticosteroids at the start of treatment has been demonstrated to reduce mortality in HIV-negative individuals but whether they are universally beneficial is unclear. Other host-directed therapies show promise but evidence for widespread use is lacking. Finally, the management of TBM within health systems is sub-optimal, with drop-offs at every stage in the care cascade.

## Introduction

Tuberculous meningitis (TBM) is an extra-pulmonary form of tuberculosis (TB) characterised by sub-acute or chronic inflammation of the meninges as a result of invasion of the sub-arachnoid space by the bacilli *M. tuberculosis*. Other forms of central nervous system (CNS) TB such as tuberculoma, cerebral abscess, and spinal TB are not categorised as TBM, even though treatment is similar. TBM most commonly affects young children and individuals with human immunodeficiency virus (HIV). In the absence of TB treatment, TBM is uniformly fatal ^1^ and even with treatment, outcomes are poor and chronic neurodisability is common. Much of the damage caused by TBM is due to host-derived inflammatory responses, yet our understanding of these processes is limited. In this review we describe the current global situation for TBM in terms of our understanding of the pathogenesis and natural history, epidemiology, diagnosis, and treatment. We also explore how TBM is managed within health systems. Challenges in the field of TBM are shown in Table 1.

**Table 1.** Challenges in tuberculous meningitis.

	Challenges	Implication
**Pathogenesis**	Mechanism of dissemination from lungs to CSF unclear Mechanisms of CSF invasion unclear	Possible preventative steps/interventions are unclear
**Natural History**	Why some individuals develop TBM unclear	Focussed prevention therapy not possible
**Epidemiology**	No good estimates of global TBM incidence No good estimates of overall mortality/ morbidity Unclear differentiation between adult and childhood TBM	Inadequate research funding, inadequate local, country and international health system investment in treatment and diagnosis of TB meningitis Management tailored for children/adults
**Diagnosis**	Inadequate ability to rule-out TBM Incomplete market penetration of the most effective tests Relatively expensive tests	Many missed cases of TBM Late diagnosis and potentially preventable long-term neurologic disability or mortality Testing unavailable in some settings due to cost
**Treatment**	Pulmonary TB treatment regimens adapted (with minimal change) to TBM Incomplete understanding of the best treatment for host immune inflammation	Likely inadequate TBM treatment regimens Uncertainty regarding dosing, optimal anti-tuberculous medications Uncertain efficacy/harm of corticosteroids in some populations
**Health Systems**	Poor community awareness Inadequate diagnostic device access	Missed diagnoses Inadequate resources committed to TBM Missed diagnoses and poor outcomes

CSF, cerebrospinal fluid; TBM, tuberculous meningitis; TB, tuberculosis.

## Pathogenesis

Primary infection occurs via inhalation of *M. tuberculosis*-containing aerosolised droplets, followed by activation of neutrophils, dendritic cells and alveolar macrophages, which engulf the mycobacteria in the terminal alveoli. Infected cells then migrate to lymphoid tissue, resulting in activation of Th1 cells and production of pro-inflammatory cytokines, with resultant inflammatory changes in the lung parenchyma and vasculature ^2^. If the mycobacteria reach the vasculature, haematogenous dissemination can occur, with the potential to invade the CNS ^3– 5^.

The pathogenesis of TBM continues to be debated. A key virulence feature of mycobacteria is the ability to invade the blood-brain and blood-cerebrospinal fluid (CSF) barriers. The invasion mechanisms are not clear, although *in vitro* and animal data suggest that *M. tuberculosis* may rearrange the actin of the layers ^4, 6^. It is also possible that a “Trojan horse” mechanism, by which *M. tuberculosis* is brought across the blood-brain barrier by infected macrophages and neutrophils, may occur ^7^. Rich and McCordock described the development of a caseating “Rich” focus in the context of TBM pathogenesis ^8, 9^. It is suggested that the Rich focus is formed via activation of microglial cells and astrocytes once the bacilli have gained access to the brain. Once formed, the Rich foci may become activated rapidly or months to years later, resulting in release of *M. tuberculosis* into the subarachnoid space, triggering an inflammatory cascade ^9^. The resulting inflammatory changes may explain some of the clinical features associated with TBM. First, peri-vascular inflammation, particularly of the middle cerebral artery, results in decreased perfusion and cerebral infarct. Second, extension of exudative material to the basal cisterns and the mid-brain leads to disruption of CSF flow, hydrocephalus and raised intracranial pressure. Third, exudates encase cranial nerves, resulting in cranial nerve palsies. Finally, expanding parenchymal tubercles may form tuberculomas and, less frequently, brain abscesses. In contrast (or in addition) to the Rich hypothesis, other researchers have suggested that bacilli reach the CSF during miliary dissemination. Donald and colleagues have proposed a pathogenic mechanism, based on more recent clinical, post mortem and epidemiological data, that articulates the central role of the Rich focus but with an additional component that includes miliary TB ^5^.

## Natural history

The risk and severity of TBM are altered by the status of the host immune response and pathogen virulence, consistent with the damage response framework ^10^. Multiple host factors such as age, HIV co-infection, Bacillus Calmette–Guérin (BCG) immunisation, malnutrition, and helminth co-infection may lead to either a deficient or exaggerated immune response to mycobacterial infection.

### Age

Several studies conducted in the pre-chemotherapy era identified children who had been exposed to TB and followed them for tuberculin skin test conversion, chest radiograph changes, clinical progression, and mycobacterial culture positivity. Marais and colleagues reviewed these studies and proposed a timetable for TB, in the absence of drug therapy ^11^. Following infection with *M. tuberculosis*, young children are at high risk of progression to TB disease including disseminated forms such as TBM. Both the risk of disease progression and risk of dissemination fall rapidly through childhood, reaching a nadir in the primary school age. In more recent cohorts of children with TBM, the median age is between two and four years ^12^. Marais and colleagues also found that children who develop miliary TB or TBM generally do so from one to four months after exposure.

### HIV

HIV co-infection is the most significant risk factor for TBM in adults and is believed to attenuate the host immune response in the CSF. The impact of HIV on the clinical presentation of TBM is debated, with some studies suggesting a higher rate of extra-meningeal disease and of TB drug resistance ^13– 16^. Other studies report that the clinical course of TBM is unaltered by HIV infection ^17, 18^.

### BCG, helminth infections and nutrition

Neonatal BCG immunization is effective in preventing childhood TB, particularly TBM and miliary TB ^19– 21^. Efficacy varies from as high as 80% near the poles, to <20% near the equator, possibly due to masking or blocking from environmental mycobacteria or modulation due to helminth infections ^20, 22, 23^. Helminth infections activate a T helper type 2 and/or regulatory T cell response and may down-regulate T helper type 1 and T helper type 17 responses to mycobacterial antigens, potentially decreasing BCG vaccine immunogenicity. Whether this is clinically important is unclear ^24– 31^. Several epidemiological, clinical and laboratory studies have demonstrated that malnutrition, a form of acquired immunodeficiency, increases susceptibility to TB. In particular, vitamin D and vitamin D receptor polymorphisms are critical for function of macrophages in the context of TBM ^32– 34^. Vitamin D deficiency was significantly more common in TBM compared to controls and pulmonary TB in an Indian study ^35^.

### Strain type

There are seven main *M. tuberculosis* lineages, each associated with varying degrees of proinflammatory host response. The modern lineage x (Beijing) is associated with disseminated extra-pulmonary disease, drug resistance, and possibly poor treatment outcomes ^36– 39^. One plausible mechanism of Beijing strain survival is a dampened IFN-γ host inflammatory response, promoting high bacterial load.

## Epidemiology

Our understanding of the global burden of TBM is poor. Because of inadequate diagnostic test performance and availability, many cases of TBM remain undiagnosed. Autopsy studies in adults with HIV have found high proportions of TB co-infection, commonly with extrapulmonary disease, including meningitis, which is frequently undiagnosed and untreated prior to death ^40^. Even those cases diagnosed may not be appropriately reported as individuals with TBM are usually diagnosed in hospitals, which in some contexts, are less likely to be reporting units for TB ^41^.

The proportion of TBM in cohorts of TB varies dramatically by the local TB prevalence, age, HIV prevalence, and whether the cohort is describing all or only hospitalized persons (which tend to have a higher proportion of TBM cases). A population-based estimate in a low TB-prevalence settings suggested that around 1% of TB cases were TBM ^42^, while a paediatric hospital-based cohort of confirmed TB in a high TB-burden setting suggested that over 10% of TB cases were TBM ^43^. Given that the World Health Organization (WHO) estimates 10.4 million new TB cases each year (of which 1 million are children), we suggest that at least 100,000 individuals develop TBM develop annually, but this figure may be much higher.

Untreated, TBM is uniformly fatal. A study from the pre-chemotherapy literature in children demonstrated that the median time to death was 19 and a half days ^44^. Even if treated, up to 20% of children will die and, of survivors, over half have severe neurodevelopmental sequalae ^45^. Mortality in HIV-infected adults treated for TBM can be as high as 60% in some cohorts, dependent on degree of immunosuppression, use of antiretroviral therapy and stage of TBM at presentation ^14, 46^. The total mortality impact of this condition is unclear, and likely underestimated.

Morbidity in TBM survivors is heavily dependent on the age at which the disease occurs and the context in which the individual is living. Survivors require extensive support from medical staff, physiotherapy, occupational therapy, pharmacists and dieticians. Many of these services are not widely available in high TB-burden settings. They also require vast commitments from family and community members, with implications for their quality of life and economic circumstances. Given that they have more potential life years ahead, children lose more disability adjusted life years than older individuals. Even in children with limited neuromotor disability following treatment, cognitive disability, attention-deficit and behavioural problems are more common with TBM survivors than with community controls ^47, 48^.

## Diagnostics

TBM remains difficult to diagnose. Clinical features such as fever, headache, neck stiffness, or a ‘typical’ CSF pattern (elevated protein, lymphocytic pleocytosis, and low glucose) cannot reliably distinguish TBM from other forms of sub-acute meningitis ^49, 50^. Neuroimaging is frequently unavailable in high TB burden contexts. Where characteristics features of TBM are present on neuroimaging, combinations of basal enhancement, tuberculomas, infarction or hydrocephalus support (but do not confirm) the diagnosis. Many cases of TBM, however, are associated with neuroimaging that does not assist with the diagnosis. Traditional tests to detect bacilli include acid fast bacilli (AFB) smear and culture. AFB smear is rapid and widely available but only 10–15% sensitive in most settings ^49, 51^. Culture is more sensitive (~50–60%) but results return too slowly to allow for clinical intervention (two-six weeks depending on the media) ^50– 53^. Death frequently occurs prior to culture results if empiric therapy has not been initiated in the interim period ^50^. Culture also requires a biosafety level three laboratory, limiting use to referral centres in many settings.

In recent years, nucleic acid amplification tests (NAATs) have been utilised for TBM diagnosis. The most notable tests are GeneXpert MTB/RIF (Xpert) and the re-engineered GeneXpert MTB/RIF Ultra (Xpert Ultra), though other commercial and ‘in-house’ NAATs have been used as well ^54^. Xpert and Xpert Ultra (Cepheid, Inc, Sunnyvale, CA, USA) are cartridge-based, rapid, fully automated polymerase chain reaction (PCR) tests. In 2013, the WHO recommended Xpert as the initial test for diagnosis of TBM based on a systematic review of 13 studies ^55– 57^. Sensitivity is generally similar to culture (50–60%), but with a run-time of two hours ^51, 53, 58^. Xpert and culture frequently detect cases that the other modality misses and Xpert’s negative predictive value is only 84–94%, meaning that while Xpert is helpful if positive, it cannot effectively rule out TBM ^51, 59, 60^. One way to maximise Xpert’s utility is to centrifuge larger volumes of CSF (>5ml), although this effect has not been universally observed ^51^. At present, access to Xpert is increasing although still not adequate ^61^.

Xpert Ultra utilizes the same instrument as Xpert with a software upgrade. WHO has recommended Xpert Ultra in place of Xpert for TB meningitis based on one study of Ugandan HIV-infected adults with suspected TBM, which found 95% sensitivity versus a composite microbiological end point, and 70% sensitivity versus consensus research case definitions ^52, 62^. This was as opposed to inferior sensitivities for both Xpert and culture versus the same reference standards ^52, 63^. A recent cohort reported 86% sensitivity for Xpert Ultra for definite TBM versus 36% for Xpert and 14% for culture ^64^. Negative predictive values remain inadequate to ‘rule-out’ TBM and the publication of additional studies of Xpert Ultra is required to fully evaluate this technology.

Although there is hope that immunodiagnostics will bridge the gap from Ultra to a definitive diagnostic tool-kit for TBM, thus far, adenosine deaminase, INF-γ release assays, and antibody tests, among others, have not yet proven adequate adjunctive tests for TBM ^54, 65^. Thus, diagnostic tests remain imperfect (though improving) and a low threshold to start empiric therapy remains a key to effective management of TB meningitis.

## Treatment

The evidence to guide treatment in TBM is limited, based mainly on experience with pulmonary TB ^66^. The first treatment option for TBM, streptomycin became available in 1946 ^67, 68^, albeit at the cost of long, painful treatment duration and poor survival ^69– 75^. Para-amino salicylic acid (PAS), introduced in the early 1950s, given in combination with streptomycin reduced resistance and mortality, even with poor CSF penetration ^72, 76– 79^. Isoniazid, with low toxicity and better CNS penetration, was introduced in 1952, whereafter outcomes improved ^76, 77, 80– 83^. Rifampicin and pyrazinamide were used in TBM treatment regimens from 1970 onwards; however, initially there was no significant reduction in mortality compared to regimens containing streptomycin, PAS and isoniazid ^76, 84– 87^.

Available treatment options for TBM are hampered by poor CSF penetration and toxicity ^76, 88^. Of the first-line TB drugs, pharmacokinetic studies have shown that isoniazid and pyrazinamide have the best CSF penetration ^88^. In children, modelling suggests that higher dosages of rifampicin may be required ^89^, while in adults, doses up to 600mg per day increases CSF penetration ^88, 90^. Ethambutol and streptomycin both have poor CSF penetration. Second-line TB drugs with good CSF penetration include ethionamide, the fluoroquinolones (levofloxacin, moxifloxacin and ofloxacin) and terizidone ^88^. Linezolid is another potential treatment option, given excellent CSF penetration ^76, 91^. Moxifloxacin up to 800mg per day has demonstrated good CSF concentration with low toxicity ^90, 92^.

Current WHO treatment guidelines for TBM recommend two months of a four-drug regimen (rifampicin, isoniazid, pyrazinamide and ethambutol) in both adults and children, followed by 7–10 months of rifampicin and isoniazid ^93, 94^. Similar dosing to pulmonary TB is advocated based on low quality evidence ( Table 2) ^88, 95^. Thus, the focus of recent TBM trials has been to optimize CNS penetration while minimizing toxicity using alternative regimens, higher doses and/or shorter treatment. Adjunctive levofloxacin (20mg/kg per day) did not demonstrate increased survival benefit among adults in one Vietnamese randomized controlled trial ^96– 98^. Regarding dosing strategies, high-dose intravenous rifampicin for the first two weeks of treatment demonstrated increased CSF drug concentration as well as significant reduction in six-month mortality ^90, 99, 100^. Similarly, a study evaluating an abbreviated course (six months in HIV-uninfected and nine months in HIV-infected children) of a high dose four-drug regimen (rifampicin 20mg/kg, isoniazid 20mg/kg, pyrazinamide 40mg/kg and ethionamide 20mg/kg) demonstrated mortality of less than 5% ^101^.

**Table 2.** World Health Organization recommended drug dosing for adults and children with tuberculous meningitis ^93, 94^.

Drug	Adult dosage and range (mg/kg)	Childhood dosage and range (mg/kg)	Maximum dosage (mg/day)
Isoniazid (H)	5 (4-6)	10 (10-15)	300
Rifampicin (R)	10 (8-12)	15 (10-20)	600
Pyrazinamide (Z)	25 (20-30)	35 (30-40)	
Ethambutol (E)	15 (15-20)	20 (15-25)	

Adjuvant immunomodulatory treatment is used in TBM to ameliorate the potentially damaging host response. A 2016 Cochrane systematic review and meta-analysis found that adjunctive corticosteroids reduce mortality in the short term but had no effect on long-term neurological disability in HIV-uninfected patients with TBM; however, the benefit of corticosteroids is unclear in HIV co-infected individuals ^102^. Corticosteroids may have a differential effect dependent on leukotriene A4 hydrolase genotype, being beneficial in those with a hyper-inflammatory genotype but detrimental in others. Use of adjunctive aspirin has been shown to be beneficial in adults with TBM ^103, 104^, with one recent study of high dose aspirin demonstrating prevention of new cerebral infarction in adults ^105^. This effect, however, has not been demonstrated in children and requires larger randomised trials in adults ^106^. Currently registered clinical trials for TBM are shown in Table 3.

**Table 3.** Currently open and registered clinical trials in tuberculous meningitis (from the ClinicalTrials.gov and ISRCTN registries).

Trial Name	Registration Number	Countries Recruiting	Target Patient Group	Study Arms	Primary Outcome	Sample Size
**Optimizing Anti-tuberculosis** **Therapy in Adults with** **Tuberculous Meningitis**	NCT03787940	China	Adults 18–65 years	Standard dose vs. high dose isoniazid for rapid NAT2 acetylators	Death or severe disability at 12 months from enrolment	338 rapid acetylators
**Retrospective Real-word** **Study of Linezolid for the** **Treatment of Tuberculous** **Meningitis**	NCT03898635	China	Adults 18 years and older	1. No linezolid in treatment regimen at any point 2. Linezolid added during the treatment course 3. Linezolid in treatment regimen from beginning	Survival within five years	n/s
**Linezolid, Aspirin and** **Enhanced Dose Rifampicin** **in HIV-TBM (LASER-TBM)**	NCT03927313	South Africa	HIV-infected adults (>18 years)	1. Standard of care drug regimen at standard of care dosages 2. Higher dosages of rifampicin and the addition of linezolid for the first 56 days of treatment 3. Higher dosages of rifampicin and the addition of linezolid and aspirin for the first 56 days of treatment	Treatment related adverse events	100
**Adjunctive Corticosteroids** **for Tuberculous Meningitis in** **HIV-infected Adults (The ACT** **HIV Trial)**	NCT03092817	Vietnam, Indonesia	HIV-infected adults (>18 years)	Dexamethasone vs. placebo	Survival 12 months post randomisation	520
**Leukotriene A4 Hydrolase** **Stratified Trial of Adjunctive** **Corticosteroids for HIV-** **uninfected Adults with** **Tuberculous Meningitis**	NCT03100786	Vietnam	HIV-uninfected adults (>18 years)	1. Open label dexamethasone for all patients with TT genotype for first 6–8 weeks treatment 2. Individuals with CT or CC genotypes randomised to dexamethasone or placebo for first 6–8 weeks treatment	All-cause mortality or new neurological event by 12 months post randomisation	640
**Optimizing Treatment to** **Improve TBM Outcomes in** **Children (TBM-KIDS)**	NCT02958709	India, Malawi	Children	1. High dose rifampicin in a standard drug regimen 2. High dose rifampicin and levofloxacin instead of ethambutol 3. Standard dose rifampicin in a standard drug regimen	Characterise the pharmacokinetic parameters of rifampicin and levofloxacin and describe outcomes and safety	120
**Harvest trial - improving** **outcomes from TB** **meningitis with high dose** **oral rifampicin**	ISRCTN15668391	Uganda, b/South Africa, Indonesia	Adults (>18 years)	Standardly dosed regimen compared to a standard drug regimen but with elevated rifampicin dosage	Six-month survival	500
**SURE: Short intensive** **treatment for children with** **tuberculous meningitis**	ISRCTN40829906	India, Uganda, Vietnam, Zambia, Zimbabwe	Children under 15 years (and over 28 days)	Factorial design: • First randomisation: standard drug regimen vs. drug regimen with high dose rifampicin and substitution of ethambutol with levofloxacin • Second randomisation: aspirin vs. placebo	First randomisation: all-cause mortality by 48 weeks Second randomisation: neurodevelopment at 48 weeks assessed using a Modified Rankin Score	400
**Improving diagnosis and** **treatment of HIV-associated** **Tuberculous meningitis**	ISRCTN42218549	Uganda	Adults (>18 years)	1. Intravenous 20mg/kg/day rifampicin for two weeks (followed by oral rifampicin 35mg/kg/day for six weeks) 2. Oral 35mg/kg/day rifampicin for eight weeks 3. Standard of care oral rifampicin (~10mg/kg/day) for eight weeks	Pharmacokinetic parameters and safety composite endpoint	60

TB mass lesions, usually in the setting of immune reconstitution inflammatory syndrome (IRIS) and optochisamic arachnoiditis, are difficult to treat and often occur near vital structures in the brainstem and spinal cord. Thalidomide, a potent TNF-α inhibitor, has demonstrated clinical benefit in both TB mass lesions and optochiasmic arachnoiditis ^107, 108^. Reports have also demonstrated a clinical role for other agents that cause TNF-α inhibition, including infliximab and IFN-γ ^109– 111^. Prophylactic pyridoxine (5–10 mg per day in children and 10 mg per day in adults) is recommended to prevent isoniazid related peripheral neuropathy ^93, 94^. Neuropathy is more severe during pregnancy, HIV co-infection, alcohol dependency, malnutrition, diabetes, chronic liver disease and renal failure. Treatment of hydrocephalus depends on the level of CSF obstruction. Communicating hydrocephalus, a common complication of TBM, can be successfully treated with one month of acetazolamide 50 mg/kg per day and frusemide 1 mg/kg per day ^112, 113^.

## Health systems

The care cascade for TBM has not been specifically investigated. For TB more generally, large gaps exist at every stage from incidence to diagnosis, from diagnosis to treatment and from treatment initiation to favourable outcome ^114– 116^. However, there are specific challenges for health systems in their management of TBM, including rehabilitation following treatment conclusion, that suggest gaps could be even larger than for TB as a whole.

Symptoms of TBM are non-specific and community sensitisation is poor. Even where sensitisation to TB has occurred, this is usually to the symptoms and signs of pulmonary TB, such as cough, weight loss, night sweats and haemoptysis ^117^. If individuals with TBM do present for evaluation, it is commonly to primary care facilities, at which staff training and experience can be limited and appropriate investigations, such as lumbar puncture or cerebral imaging, are often unavailable. Multiple presentations to healthcare workers are common and are associated with delayed diagnosis and treatment initiation ^118, 119^. Referral from primary care to hospital can lead to further delays and frequently incur costs to patients and families. These delays can lead to clinical deterioration ^120^.

Even when an individual presents to a hospital for evaluation, the availability of Xpert is variable globally ^61^. Although AFB smear is widely available, it has limited sensitivity and culture, if available, returns results in a timescale that is not clinically useful. Although drug therapy is usually provided freely by national TB programmes and is available at peripheral care settings, the specialist services required to appropriately manage TBM, such as paediatrics, neurology, neurosurgery and rehabilitation, are usually only available in specialist centres in larger urban areas and frequently incur costs for patients.

Surveillance data for TBM is limited, with cases rarely reported if patients die before starting treatment. Many patients with TBM are cared for in hospitals for most of their illness and given that many hospitals are not TB-reporting units, these cases are not reflected in regional, national or global reports. Disaggregation of data occurs to the level of pulmonary TB and extrapulmonary TB, without specifying the site of disease. Some electronic registers allow the recording of International Classification of Diseases 10 ^th^ revision (ICD-10) codes, but these are often poorly completed.

## Conclusions

TBM is the most severe form of TB and is associated with high mortality and morbidity. Our understanding of the global burden is limited but it is likely that most cases are not diagnosed and appropriately treated. At least 100,000 cases are likely to occur each year. The current diagnostic tools are largely inadequate, and treatment is usually started only once substantial neurological damage has occurred. Treatment, both anti-mycobacterial and anti-inflammatory, require further investigation and optimisation, and improvements at every stage along the care cascade are urgently needed. 

## Data availability

No data are associated with this article.
