# Supplementary material for: The current global situation for tuberculous meningitis: epidemiology, diagnostics, treatment and outcomes
Source: Wellcome Open Res. 2019 Nov 5;4:167. [Version 1] doi: 10.12688/wellcomeopenres.15535.1 (PMC7029758; doi:10.12688/wellcomeopenres.15535.1)
Supplement: Peer review history

# Peer review: Reviewer response for version 1

**DOI:** 10.21956/wellcomeopenres.17007.r37823

**Published:** 2020-02-17

**Authors:** Tamilarasu Kadhiravan

**Affiliations:** 1 Department of Medicine, Jawaharlal Institute of Postgraduate Medical Education and Research, Puducherry, Puducherry, India

In this article, Seddon *et al*. have provided a comprehensive overview of the pathogenesis, epidemiology, and diagnostics of tuberculous meningitis. Further, this article traces the historical progress made in the treatment of tuberculous meningitis, and also provides a succinct summary of the recently concluded as well as ongoing clinical trials of intensified/adjunctive treatments for tuberculous meningitis. Overall, this is a well written article, and it provides a contextual background for the other articles in the collection.

 I have two suggestions. First, one of the important barriers to early diagnosis of tuberculous meningitis could be the (incorrect) notion that tuberculous meningitis is a subacute disease process. Contrary to this notion, nearly half the patients with tuberculous meningitis seen in many settings have less than 2 weeks of symptoms at presentation. However, clinicians still fail to consider tuberculous meningitis in such patients with less than 2 weeks of symptoms.

 Second, I am not sure how many of the national TB control programmes worldwide provide dexamethasone free-of-cost to tuberculous meningitis patients. My suspicion is that current health systems are unable to reliably deliver the only effective adjunctive treatment available to treat tuberculous meningitis.

I confirm that I have read this submission and believe that I have an appropriate level of expertise to confirm that it is of an acceptable scientific standard.

# Peer review: Reviewer response for version 1

**DOI:** 10.21956/wellcomeopenres.17007.r36945

**Published:** 2019-12-09

**Authors:** Kiran T. Thakur

**Affiliations:** 1 Department of Neurology, Columbia University Irving Medical Center, New York City, NY, USA

Experts in TB and TB meningitis in the Tuberculous Meningitis International Research Consortium provide an overview on the global situation for tuberculous meningitis and discuss current knowledge around epidemiology, diagnostic testing and treatment for TB meningitis. The authors provide a well-written article around the basic aspects of TBM which provides an important overview on the topic, including knowledge gaps in our fundamental understanding of TBM pathogenesis, diagnosis and treatments. Though much of the review of data has been previously published, authors provide important novel aspects including implications of our lack of knowledge in key aspects of TBM, and aspects of healthcare system infrastructure in current TBM care.

 The authors discuss the debate in TBM pathogenesis including historical work by Roch and McCordock. Additional commentary on miliary TB pathogenesis would be useful to the readership.

 The natural history of TBM is discussed around host factors. The discussion focuses on risk factors including age, HIV, BCG vaccine, helminth infections, nutrition and strain type though importantly does not mention other causes of immunocomprised state including immunomodulatory treatments and possible environmental factors which are of growing importance globally (overcrowding in inner city environments, pollution, etc). A brief discussion on the immunological aspects which may children particularly susceptible to disseminated disease would also be educational to the reader. The discussion of strain type as a risk factor is important, though only touched upon very briefly in this article and worthy of expansion.

 The unknowns in terms of current epidemiology of TBM is also focused upon. It would be worthwhile for authors to summarize past epidemiological data regionally, and also note ongoing studies on epidemiology of TBM. An important milestone would be a multicenter international study on epidemiology of TBM, and the consortium may be planning such work.

 Epidemiological studies are limited by current diagnostics in TBM, and the authors importantly note the limited availability of gene xpert and gene xpert ultra. It would be important to identify where this testing is actually available and the barriers to implementation of such testing at the healthcare system level in those where the testing does not exist.

 The treatment discussion is nicely delineated, with a historical and current perspective discussion. Missing from the discussion is commentary on treatment in the pediatric population and treatment initiation of antiretroviral medications in patients with TBM, both challenges to those at the bedside treating such patients. It would be worthwhile to expand the treatment section to include supportive care given the importance of neurointensive care efforts including ICP monitoring, treatment of hyponatremia, when to consider shunting etc.  

 The healthcare systems section of the paper is important and novel and would benefit perhaps from a visual of the current system at the primary, secondary and tertiary care health systems levels and major knowledge gaps/policy gaps in clinical practice. 

 Expansion of Table one is also important in terms of future directions beyond implications of challenges in TBM--how do we study and fill this knowledge gap is essential in TBM. 

 Overall, while the article does not provide new data on TBM, the review provides an important summary on our current state of knowledge, with an emphasis on knowledge gaps. I applaud the authors for their work on this important disease, and look forward to further fruitful work from the consortium.

I confirm that I have read this submission and believe that I have an appropriate level of expertise to confirm that it is of an acceptable scientific standard.
